# Adherence to statins and its impact on clinical outcomes: a retrospective population-based study in China

**DOI:** 10.1186/s12872-020-01566-2

**Published:** 2020-06-10

**Authors:** Boya Zhao, Xiaoning He, Jing Wu, Shu Yan

**Affiliations:** 1 School of Pharmaceutical Science and Technology, Tianjin University, No 92 Weijin Road, Nankai District, Tianjin, 300072 China; 2 Pharmacy Department, Nankai Hospital, No.122 Sanwei Road. Nankai District, Tianjin, 300072 China

**Keywords:** Statin adherence, Clinical outcomes, Cardiovascular disease, Primary prevention, Secondary prevention

## Abstract

**Background:**

While the benefit of adherence to statins on clinical outcomes has been proved, this benefit may be heterogeneous among patients who initiated statins for primary or secondary prevention purpose. This study aimed to investigate the impact of statin adherence on clinical outcomes among patients who initiated statins for primary and secondary prevention in China.

**Methods:**

Adult patients in Tianjin Urban Employee Basic Medical Insurance database who initiated ≥2 prescriptions of statins from 2012 through 2013 were included and grouped into primary and secondary prevention subgroups according to their cardiovascular diseases (CVD) history during the prior 12-month baseline period. Proportion of days covered (PDC) was used to measure statin adherence in the initial 12-month follow-up. Clinical outcomes were measured by the incidence of major adverse cardiovascular events (MACE) during the 13th–24th months follow-up, and were compared between the patients with PDC ≥ 0.5 and patients with PDC < 0.5 using Cox regression models in primary and secondary prevention subgroups. Sensitivity analyses were conducted in propensity score matched groups.

**Results:**

99,655 patients were finally included. The mean (SD) PDC was 0.19 (0.15) in primary prevention subgroup (*N* = 34,372), with 5.4% patients had PDC ≥ 0.5. The patients with PDC ≥ 0.5 had a 37% reduced risk of MACE compared with patients with PDC < 0.5 (Unadjusted incidence rate of MACE: 1.1% vs. 1.4%; all-adjusted HR = 0.63; 95% CI, 0.41–0.98). While, no significant difference was observed in the secondary prevention subgroup (*N* = 65,283) between patients with PDC ≥ 0.5 and patients with PDC < 0.5 (Unadjusted incidence rate of MACE: 4.6% vs. 2.8%; all-adjusted HR = 1.08, 95% CI, 0.92–1.28). These findings were confirmed by the sensitivity analyses in propensity score matched groups.

**Conclusions:**

Statin adherence was very poor in China, and statin adherence is associated with decreased risk of MACE in patients for primary prevention, while further exploration is needed for secondary prevention.

## Background

HMG-CoA reductase inhibitors (statins) are recommended as the first-line of lipid-lowering drug therapy in the primary and secondary prevention of cardiovascular events [1–3]. Long-term usage of statins can effectively lower the plasma levels of low-density lipoprotein cholesterol and total cholesterol, and reduce the subsequent risk of major adverse cardiovascular events (MACE) [1, 4–7]. However, adherence to statins in real-world clinical practice is known to be suboptimal. It was reported that only 35% ~ 70% patients were adherent to statins with a ≥ 80% proportion of days covered (PDC) or medication possession ratio (MPR) in developed countries including UK, Italy and Finland [8–13], and statin adherence could be even poorer in developing countries such as China.

Poor adherence to statins therapy has been reported to have real clinical consequences. Systematic reviews and meta analyses have shown that low levels of statin adherence (PDC or MPR < 80%) were associated with increased risk of fatal and nonfatal cardiovascular events and all-cause mortality [13, 14]. However, the impact of poor adherence on clinical outcomes may be different between statin users for primary prevention (those who don’t have prior cardiovascular disease, CVD) and secondary prevention (those who have prior CVD) of CVD. Findings in Canada suggested that good statin adherence (PDC ≥ 0.8 or 0.9) was associated with 18 ~ 26% risk reduction of adverse clinical outcomes among patients without CVD [15–17], while the corresponding risk reduction was 13% for patients who already had CVD [18]. Similar findings were also found in related studies conducted in the US [19, 20]. However, the estimates could be varied across different studies as the result of different population, study design, study years, etc. No study has compared this risk reduction between patients for primary or secondary prevention within the same study. Related studies based on Asian population were also very limited [8, 12, 21].

This study focused on all new statin users, but separated them into 2 subgroups according to whether a patient initiated statin for primary or secondary prevention of CVD purpose, and aimed to investigate the adherence to statins and its impact on clinical outcomes in each subgroup.

## Methods

### Data sources

Data were obtained from the Urban Employee Basic Medical Insurance (UEBMI) claims of Tianjin from 2011 through 2015, through a formal request to Tianjin Municipal Human Resources and Social Security Bureau for research purposes. The UEBMI system is one of the basic medical insurance systems in People’s Republic of China. Mandatory participation is planned for all employees in both public and private companies and the UEBMI covers both employees and retirees. By 2015, the Tianjin UEBMI system covered about 5.22 million enrollees, which represented 50.8% of registered Tianjin residents [22]. The analytical sample in this study was a random sample of 30% of all enrollees of the Tianjin UEBMI. The extracted data included patient-level demographic information, medical claims of inpatient and outpatient services, prescription claims (quantity, strength, date of prescription, etc.), and related medical and medication costs. The Tolerability and Ethics Committee at the School of Pharmaceutical Science and Technology in Tianjin University waived the requirement of ethics approval for the current study as this was a retrospectively observational study using the de-identified medical claims data.

### Study population

Adult patients (≥18 years of age) initiated ≥2 prescriptions of statins (atorvastatin, simvastatin, rosuvastatin, fluvastatin, pravastatin, pitavastatin, lovastatin, or amlodipineatrovastatin) from January 1, 2012 through December 31, 2013 (identification period) were identified. The index date was defined as the date of patient’s first statin prescription in the identification period. Patients who were not continuously enrolled or had any diagnosis of malignant disease during the 12 months pre-index (baseline) and 24 months post-index (follow-up) period were excluded. Patients were further excluded if they had any statin prescription at baseline (not new statin user), have been prescribed ≥2 different statins at the index date, or experienced adverse clinical outcomes including myocardial infarction (MI), stroke, and all-cause death during the initial 12-month follow-up period.

Included patients were then grouped into CVD primary and secondary prevention subgroups according to whether they had any evidence of CVD in baseline. To be specific, patients who had any diagnosis of coronary heart disease (CHD, ICD-10 codes I20-I25), cerebrovascular disease (ICD-10 codes I60-I69, G45, G46), atherosclerosis (ICD-10 code I70), aneurysm (ICD-10 code I71), heart failure (ICD-10 code I50), or had any surgery record of coronary artery bypass grafting (CABG), percutaneous coronary intervention (PCI), carotid endarterectomy (CEA), thrombolytic therapy in baseline were assigned to secondary prevention subgroup. Patients who had no evidence of above disease or surgery record were assigned to primary prevention subgroup. All the disease records were identified by ICD-10 codes supplemented by Chinese descriptions of diagnoses, while all the surgery records were identified by related procedure codes in UEBMI claims system.

Patients’ baseline characteristics were captured during the 12-month baseline period including patient demographics (age, gender), type of initial statin at the index date, Charlson Comorbidity Index (CCI), medical history (comorbidities including hypertension, dyslipidemia, diabetes mellitus, chronic kidney disease (CKD), and CVD-related diseases and surgeries), baseline medication use (antiplatelet agents, antihypertensive agents, hypoglycemic agents, lipid-lowing agents except statins), baseline health care resource utilization and all-cause direct medical costs.

### Assessment of adherence

Adherence to statins was estimated as the proportion of days covered, calculated by the number of days covered by statins divided by the observation time interval, which was the initial 365 follow-up days in this study. Prescriptions were all picked up from pharmacies inside and outside the hospitals. For statins prescribed during a hospitalization, the days of hospitalization were counted as days covered by statins. For statins prescribed during an outpatient visit, the days covered by statins were calculated by the dispensed quantity and daily strength. Overlapping days of statin therapy between statin prescriptions were excluded. Patients with ≥0.8 of PDC, the commonly used cutoff, were deemed adherent according to previous studies [10, 11, 19]. The alternative cutoff point of 0.5 was also tried considering the expected very poor statin adherence in the studied population.

### Clinical outcomes

The clinical outcomes of interest were examined by the existence of major adverse cardiovascular events during the 13th–24th month follow-up period, which was a composite end point of MI (ICD-10 codes I21-I22), stroke (ICD-10 codes I60-I64), and all-cause death. MI and stroke were identified by ICD-10 codes only in inpatient claims.

### Statistical analysis

Descriptive analyses of patients’ demographic and baseline characteristics were conducted. The adherence was estimated in both primary and secondary prevention subgroups. To describe the trend of the adherence in the initial 12-month follow-up period, the adherence to statins was also measured monthly to present the proportions of patients with PDC ≥ 0.8, 0.8 > PDC ≥ 0.6, 0.6 > PDC ≥ 0.4, 0.4 > PDC ≥ 0.2, 0.2 > PDC in stacked area chart. After excluding patients with MACE in the initial 12-month follow-up period, patients in each subgroup were then divided into adherent (PDC ≥ 0.8 or PDC ≥ 0.5) and non-adherent (PDC < 0.8 or PDC < 0.5) patients according to their PDC values in the initial 12-month follow-up period. The incidence of MACE was described in Kaplan-Meier curve and the occurrence of MACE was compared between patients with PDC ≥ 0.8/0.5 and patients with PDC < 0.8/0.5 in each subgroup.

Unadjusted, age-sex adjusted, and all-adjusted hazard ratios (HR) were calculated to identify the association between statin adherence and risk of MACE in each subgroup, using univariate and multivariate Cox proportional hazard models. Demographics, initial statins type, baseline CCI, baseline medical history, baseline medication use, baseline health care utilization and direct medical costs were included as potential confounders in the multivariate analyses.

Sensitivity analysis using a matched pair design with propensity score matching (PSM) was conducted. Logistic regression models were used to generate propensity score for each patient considering all potential confounders mentioned above. 1:1 nearest-neighbor matching method was used to form match pairs of patients with PDC ≥ 0.8/0.5 and patients with PDC < 0.8/0.5 in primary and secondary prevention subgroups. Cox regression models were further conducted.

Statistics analysis was conducted using STATA 13.0 (StataCorp LP, College Station, TX, USA). The significance level was set as two-sided α < 0.05.

## Results

### Baseline characteristics

**Fig. 1:**
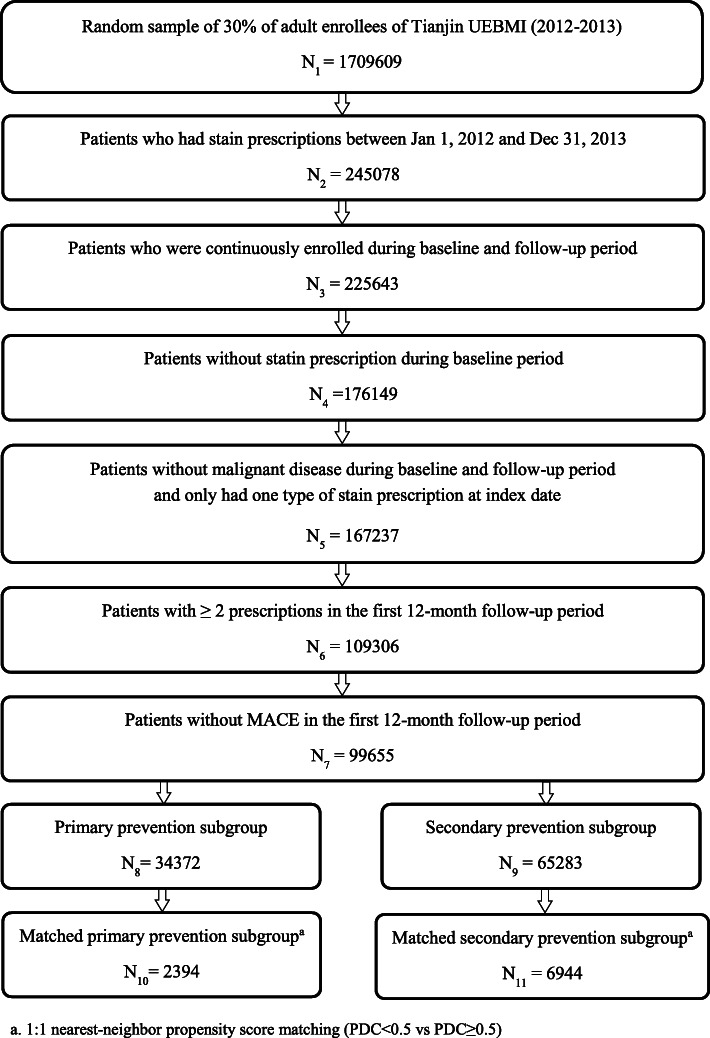
Flowchart of study inclusion and exclusion criteria

As shown in Fig. 1, a total of 99,655 eligible patients were identified, with 34,372 (34.5%) in primary prevention subgroup and 65,283 (65.5%) in secondary prevention subgroup. The demographics and baseline characteristics are presented in Table 1. In primary prevention subgroup, the mean (standard deviation (SD)) age of the patients was 50.8 (12.7), with 50.0% being female. The mean (SD) CCI among the primary prevention subgroup was 0.5 (0.8), and 36.3% patients had hypertension, 18.7% had diabetes mellitus, 16.5% had dyslipidemia. About 32.4% patients in primary prevention group used antihypertensive agents in baseline, 16.1% used hypoglycemic agents and 9.4% used antiplatelet agents. Atorvastatin was the most common used statins at index date (44.2%), followed by simvastatin (32.3%), rosuvastatin (12.4%), fluvastatin (8.6%). In baseline, only 0.4% of the patients in primary prevention subgroup experienced ≥1 hospitalization, and the mean (SD) baseline total cost was CNY 2894 (6531).

**Table 1:** Baseline characteristics of the primary and secondary prevention subgroups

Baseline characteristics	Primary prevention***N*** = 34,372	Secondary prevention***N*** = 65,283	***P*** value
Demographic characteristics			
Mean age, mean (SD)	50.8 (12.7)	59.0 (11.2)	< 0.001^a^
Age group, n (%)			
(−, 45]	11,392 (33.1)	6792 (10.4)	
(45, 55]	9709 (28.2)	17,158 (26.3)	
(55, 65]	9515 (27.7)	24,279 (37.2)	
(65, 75]	2837 (8.3)	11,455 (17.5)	
(75, +)	919 (2.7)	5599 (8.6)	
Female, n (%)	17,195 (50.0)	30,285 (46.4)	< 0.001^b^
CCI, mean (SD)	0.5 (0.8)	1.2 (1.2)	< 0.001^a^
Comorbidities, n (%)			
Hypertension	12,485 (36.3)	53,564 (82.0)	< 0.001^b^
Dyslipidemia	5672 (16.5)	29,654 (45.4)	< 0.001^b^
Diabetes mellitus	6429 (18.7)	26,688 (40.9)	< 0.001^b^
Chronic kidney disease	1099 (3.2)	5503 (8.4)	< 0.001^b^
Prior medication use, n (%)			
Antiplatelets	3215 (9.4)	31,171 (47.7)	< 0.001^b^
Antihypertensives	11,125 (32.4)	51,142 (78.3)	< 0.001^b^
Hypoglycemics	5537 (16.1)	23,744 (36.4)	< 0.001^b^
Lipid-lowing agents (except statins)	902 (2.6)	5087 (7.8)	< 0.001^b^
Index statin type, n (%)			
Atorvastatin	15,197 (44.2)	29,710 (45.5)	< 0.001^b^
Fluvastatin	2954 (8.6)	5567 (8.5)	0.720^b^
Rosuvastatin	4277 (12.4)	8430 (12.9)	0.035^b^
Simvastatin	11,099 (32.3)	20,327 (31.1)	< 0.001^b^
Others	845 (2.5)	1249 (2.0)	< 0.001^b^
All-cause resource utilization and cost			
Total direct medical cost (CNY), mean (SD)	2894 (6531)	8689 (13006)	< 0.001^a^
Number of outpatient visits, mean (SD)	11.4 (16.1)	32.1 (31.4)	< 0.001^a^
Any hospitalization, n (%)	1245 (0.4)	9047 (13.9)	< 0.001^b^

*Abbreviations*: *CCI* Charlson Comorbidity Index, *SD* Standard deviation

Notes: ^a^ Student’s t-test, ^b^ Chi-square test

Compared with primary prevention subgroup, the patients in secondary prevention subgroup were older (mean (SD) age: 59.0 (11.2) years vs. 50.8 (12.7) years; *P* < 0.001) and had more comorbidities (mean (SD) CCI: 1.2 (1.2) vs. 0.5 (0.8); *P* < 0.001). Patients in secondary prevention subgroup were also more likely to use antiplatelet, antihypertensive, hypoglycemic, and other lipid-lowing agents in baseline and had higher average total costs in baseline period (All *P* < 0.001).

### Adherence to statins

**Fig. 2:**
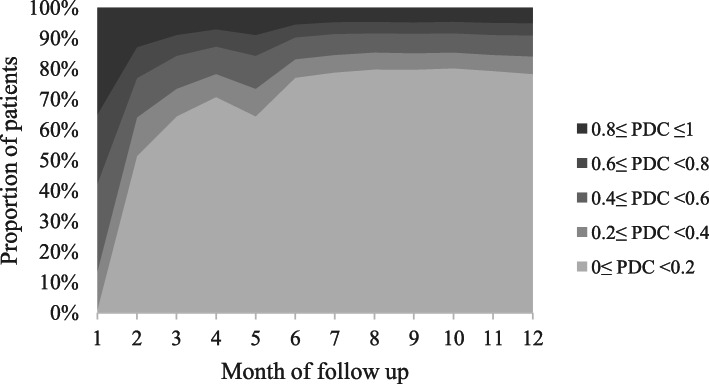
Proportion of new statin users (*N* = 109,306) with different PDC in the first 12-month follow-up period

For 109,306 identified new stain users, the mean (SD) PDC was 0.20 (0.16) during the initial 12-month follow-up period, and 0.8% patients had PDC ≥ 0.8 (Table 2). The detailed descriptive data was shown in Fig. 2, which suggested that the statin adherence was poor in the first follow-up month (PDC ≥ 0.8: 34.9%; PDC ≥ 0.5: 63.7%), got much worse in the second (PDC ≥ 0.8: 12.8%; PDC ≥ 0.5: 32.4%) and third follow-up month (PDC ≥ 0.8: 8.9%; PDC ≥ 0.5: 19.1%), and then decreased steadily or kept constant during the remaining follow-up months.

**Table 2:** Statin adherence during the initial 12-month follow-up period of the all new statin users, primary and secondary prevention subgroups

	All new statin users ***N*** = 109,306	Primary prevention subgroup ***N*** = 34,372	Secondary prevention subgroup ***N*** = 65,283
PDC, mean (SD)	0.20 (0.16)	0.19 (0.15)	0.19 (0.16)
Subgroups with 0.2 as interval, n (%)			
0 ≤ PDC < 0.2	71,580 (65.5)	22,899 (66.6)	43,323 (66.4)
0.2 ≤ PDC < 0.4	25,652 (23.5)	7879 (22.9)	15,273 (23.4)
0.4 ≤ PDC < 0.6	8510 (7.8)	2637 (7.7)	4753 (7.3)
0.6 ≤ PDC < 0.8	2701 (2.5)	769 (2.2)	1437 (2.2)
0.8 ≤ PDC ≤ 1.0	865 (0.8)	188 (0.6)	497 (0.8)
Subgroups with 0.5 as interval, n (%)			
0 ≤ PDC < 0.5	102,820 (94.1)	32,507 (94.6)	61,768 (94.6)
0.5 ≤ PDC ≤ 1.0	6486 (5.9)	1865 (5.4)	3515 (5.4)

Notes: All new statin users: statin users including patients with MACE in the initial 12-month follow-up period. Primary/Secondary prevention subgroup: Final samples after excluding patients with MACE in the initial 12-month follow-up period

During the initial 12-month follow-up period, the mean (SD) PDC were 0.19 (0.15) and 0.19 (0.16) in primary (*N* = 34,372) and secondary (*N* = 65,283) prevention subgroups, respectively. Around 66% of patients had PDC < 0.2 in both subgroups (Table 2). In primary prevention subgroup, only 0.6% patients had PDC ≥ 0.8 and about 5.4% patients had PDC ≥ 0.5 during the initial 12-month follow-up period. In secondary prevention subgroup, the corresponding proportions of patients with PDC ≥ 0.8 and PDC ≥ 0.5 were 0.8 and 5.4%, respectively. Considering the very limited sample size of patients with PDC ≥ 0.8, 0.5 was finally chosen as the cutoff point of PDC in the following analyses.

### Clinical outcomes

**Fig. 3:**
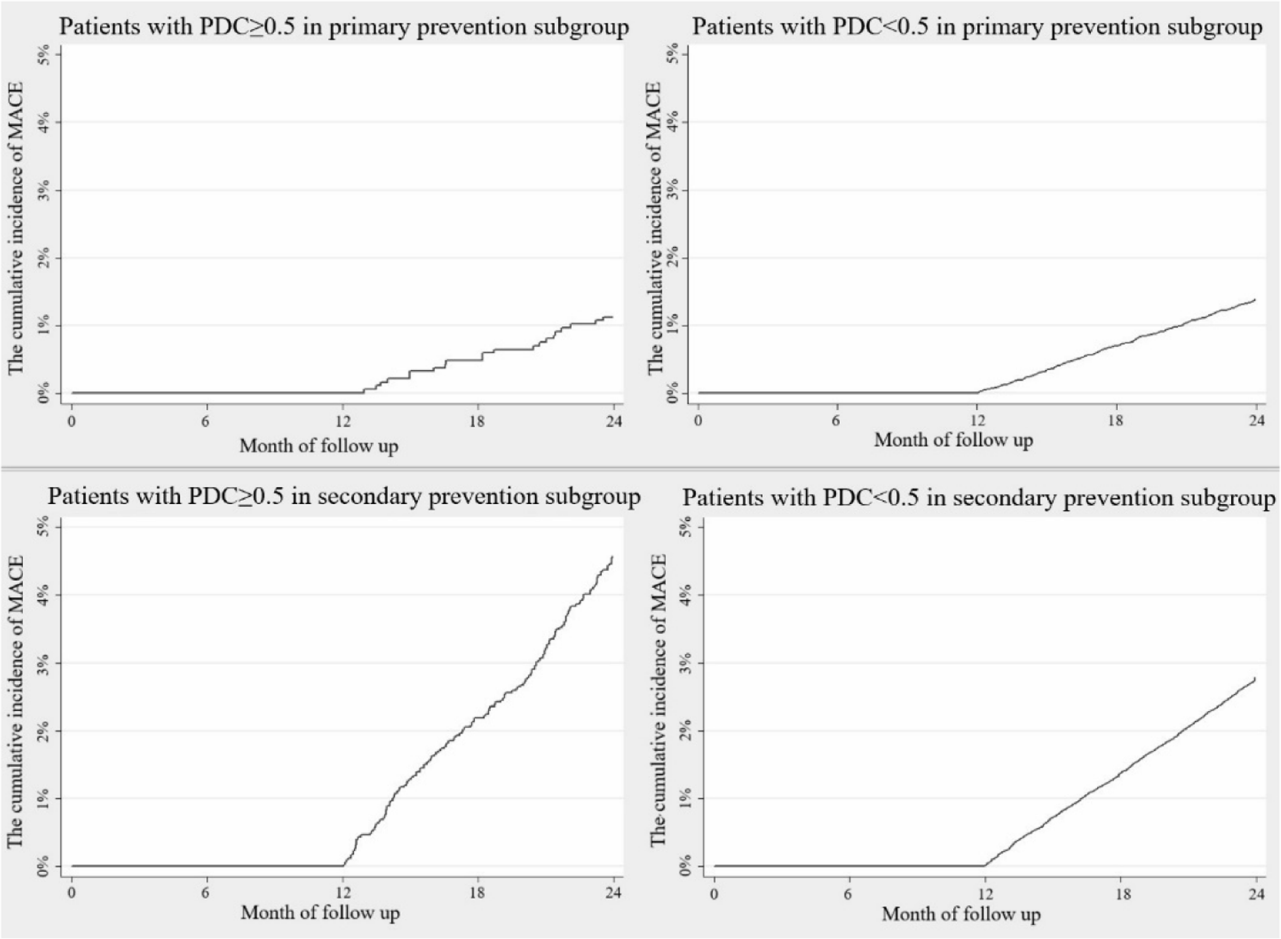
The cumulative incidence of MACE in primary (*N* = 34,372) and secondary (*N* = 65,283) prevention subgroups

**Fig. 4:**
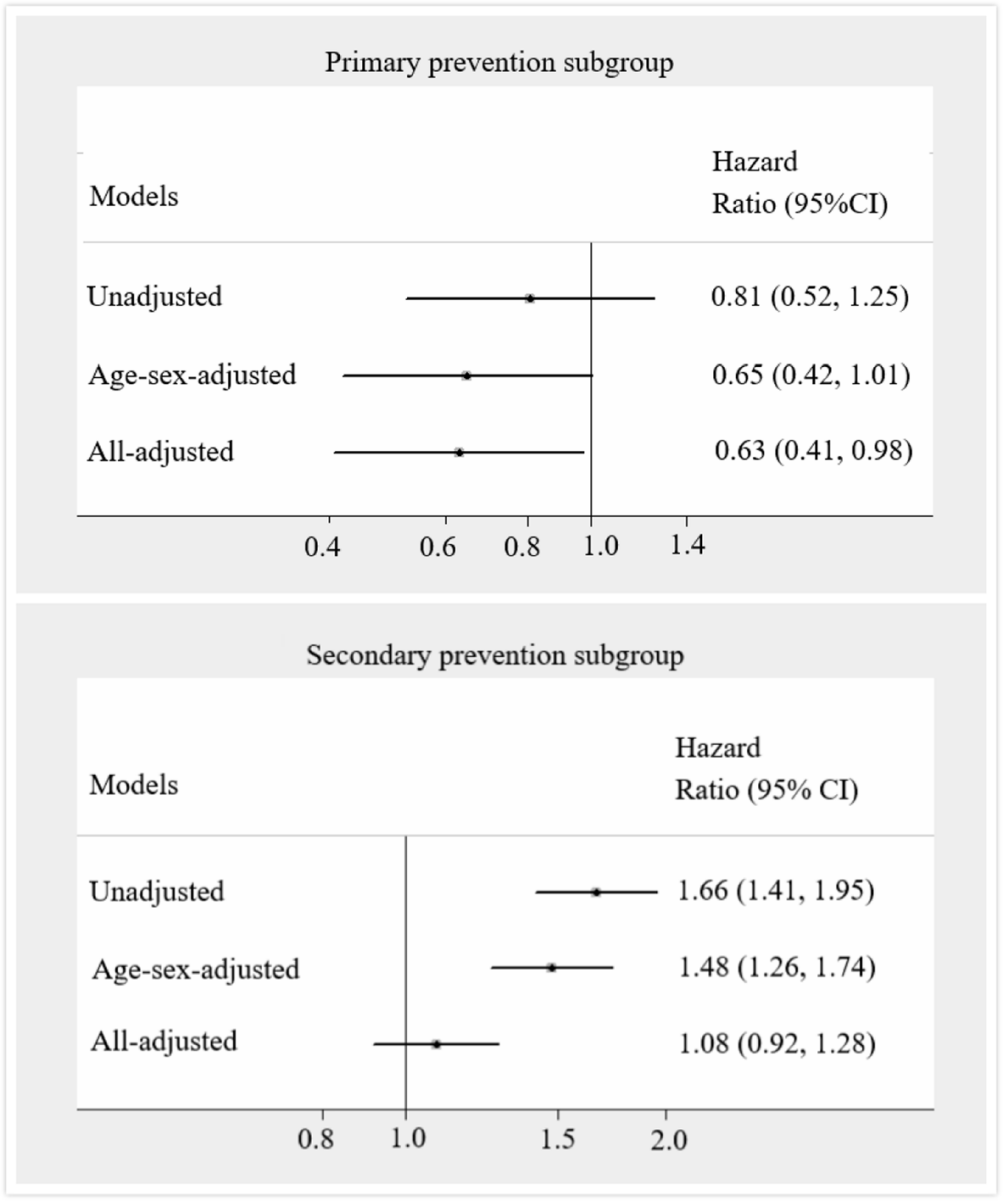
Unadjusted and adjusted risks of MACE between patients with PDC ≥ 0.5 and PDC < 0.5 in primary (*N* = 34,372) and secondary (*N* = 65,283) prevention subgroups

Patients were further divided into patients with PDC ≥ 0.5 and patients with PDC < 0.5 in primary and secondary prevention subgroup. Baseline characteristics, the occurrence and risk of MACE between patients with PDC ≥ 0.5 and patients with PDC < 0.5 were compared in primary (Table 3, Fig. 3, Fig. 4) and secondary prevention (Table 4, Fig. 3, Fig. 4) subgroups.

**Table 3:** Baseline characteristics, occurrence of MACE between patients with PDC ≥ 0.5 and PDC < 0.5 in primary prevention subgroup

	Primary prevention subgroup ***N*** = 34,372		
	Patients with PDC ≥ 0.5 ***N*** = 1865	Patients with PDC < 0.5 ***N*** = 32,507	***P*** value
**Baseline characteristics**			
Demographic characteristics			
Mean age, mean (SD)	53.7 (12.3)	50.6 (12.7)	< 0.001^a^
Female, n (%)	839 (45.0)	16,356 (50.3)	< 0.001^b^
CCI, mean (SD)	0.4 (0.7)	0.5 (0.8)	< 0.001^b^
Comorbidities, n (%)			
Hypertension	553 (29.7)	11,932 (36.7)	< 0.001^b^
Dyslipidemia	196 (10.5)	5476 (16.8)	< 0.001^b^
Diabetes mellitus	362 (19.4)	6067 (18.7)	0.421^b^
Chronic kidney disease	50 (2.7)	1049 (3.2)	0.192^b^
All-cause resource utilization and cost			
Total direct medical cost (CNY), mean (SD)	3007 (9164)	2887 (6347)	0.440^a^
Number of outpatient visits, mean (SD)	10.3 (19.8)	11.5 (15.8)	0.002^a^
Any hospitalization, n (%)	59 (3.2)	1186 (3.6)	0.276^b^
**MACE in the 13th–24th months**			
Patients with MACE, n (%)	21 (1.1)	452 (1.4)	0.340^b^
Mean number of MACE in patients with MACE, mean (SD)	1.2 (0.5)	1.3 (0.8)	0.571^a^
Days to the first MACE since index, mean (SD)	557.2 (105.6)	548.5 (104.1)	0.708^a^

Notes: ^a^ Student’s t-test, ^b^ Chi-square test

**Table 4:** Baseline characteristics, occurrence of MACE between patients with PDC ≥ 0.5 and PDC < 0.5 in secondary prevention subgroup

	Secondary prevention ***N*** = 65,283		
	Patients with PDC ≥ 0.5 ***N*** = 3515	Patients with PDC < 0.5 ***N*** = 61,768	***P*** value
**Baseline characteristics**			
Demographic characteristics			
Mean age, mean (SD)	60.5 (10.5)	59.0 (11.3)	< 0.001^a^
Female, n (%)	1494 (42.5)	33,504 (54.2)	< 0.001^b^
CCI, mean (SD)	1.5 (1.4)	1.2 (1.2)	< 0.001^b^
Comorbidities, n (%)			
Hypertension	2866 (81.5)	50,698 (82.1)	0.416^b^
Dyslipidemia	1611 (45.8)	28,043 (45.4)	0.617^b^
Diabetes mellitus	1622 (46.1)	25,066 (40.6)	< 0.001^b^
Chronic kidney disease	322 (9.2)	5181 (8.4)	0.109^b^
All-cause resource utilization and cost			
Total direct medical cost (CNY), mean (SD)	14,627.6 (21,026.1)	8351.5 (12,308.9)	< 0.001^a^
Number of outpatient visits, mean (SD)	39.9 (43.8)	31.7 (30.5)	< 0.001^a^
Patients had hospitalization record, n (%)	814 (23.2)	8233 (13.3)	< 0.001^b^
**MACE in the 13th–24th months**			
Patients with MACE, n (%)	161 (4.6)	1718 (2.8)	< 0.001^b^
Mean number of MACE in patients with MACE, mean (SD)	1.2 (0.4)	1.3 (0.7)	0.095^a^
Days to the first MACE since index, mean (SD)	552.8 (112.4)	549.3 (107.3)	0.694^a^

Notes: ^a^ Student’s t-test, ^b^ Chi-square test

In primary prevention subgroup, patients with PDC ≥ 0.5 were older (53.7 (12.3) vs. 50.6(12.7), *P* < 0.001) and less likely to be female (45.0% vs. 50.3%, *P* < 0.001) or have comorbidities including hypertension and dyslipidemia (Table 3). The cumulative incidence of MACE was shown in Fig. 3. Although there is no significant difference in risk of MACE between patients with PDC ≥ 0.5 and patients with PDC < 0.5 in unadjusted (incidence rate of MACE: 1.1% vs. 1.4%, *P* = 0.340; HR = 0.81, 95% CI: 0.52–1.25) and age-sex-adjusted (HR = 0.65, 95% CI: 0.42–1.01) Cox regression models, a significant risk reduction was found in patients with PDC ≥ 0.5 after adjusting all confounders (HR = 0.63, 95% CI: 0.41–0.98) (Fig. 4). During the 13th–24th months follow-up, stroke is the most common MACE both in patients with PDC ≥ 0.5 and patients with PDC < 0.5. The mean (SD) number of MACE were 1.2 (0.5) in patients with PDC ≥ 0.5 with MACE in primary prevention subgroup and 1.3 (0.8) in patients with PDC < 0.5 (*P* = 0.571). The average time to the first MACE since index for patients with PDC ≥ 0.5 with MACE in primary prevention group was 557.2 days, while in patients with PDC < 0.5 the average time was 548.5 days.

Similar analyses were performed in secondary prevention subgroup (Table 4, Fig. 3, Fig. 4). Patients with PDC ≥ 0.5 in secondary prevention were older (60.5 (10.5) vs. 59.0 (11.3), *P* < 0.001) compared with patients with PDC < 0.5. 4.6% (*N* = 161) of patients with PDC ≥ 0.5 had MACE during the 13th–24th months follow-up, while the corresponding rate is 2.8% for patients with PDC < 0.5 (*P* < 0.001). However, no significant relationship was observed between statin adherence and MACE in all-adjusted Cox regression model (HR = 1.08, 95% CI: 0.92–1.28) in secondary prevention subgroup.

Sensitivity analysis based on matched cohorts verified the findings from the main analysis (Table 5). After matching, PDC ≥ 0.5 were associated with reduced risk of MACE in primary prevention subgroup (Unadjusted incidence rate of MACE: 0.8% vs. 2.1%; All-adjusted: HR = 0.38, 95% CI: 0.18–0.80), while no significant relationship between statin adherence and risk of MACE was found in secondary prevention subgroup (Unadjusted incidence rate of MACE: 4.4% vs. 4.0%; All-adjusted: HR = 1.14, 95% CI: 0.91–1.44).

**Table 5:** MACE in the 13th–24th months follow-up period between patients with PDC ≥ 0.5 and PDC < 0.5 in the matched primary and secondary prevention subgroups after PSM

Subgroups	Adherence	Number of patients	Patients with MACE	HR (95%CI)		
				Unadjusted	Age-sex- adjusted	All-adjusted
Primary prevention subgroup	PDC ≥ 0.5	1197	10 (0.8%)	0.40 (0.19, 0.83)	0.39 (0.19, 0.81)	0.38 (0.18, 0.80)
	PDC < 0.5	1197	25 (2.1%)	1(ref.)	1(ref.)	1(ref.)
Secondary prevention subgroup	PDC ≥ 0.5	3472	152 (4.4%)	1.09 (0.86, 1.37)	1.10 (0.88, 1.39)	1.14 (0.91, 1.44)
	PDC < 0.5	3472	140 (4.0%)	1(ref.)	1(ref.)	1(ref.)

Abbreviation: *PSM* Propensity Score Matching

## Discussion

Different from the previous studies which focused all statin users as a whole, this study was conducted among statin users for primary and secondary prevention separately, which presented new evidence on the impact of statin adherence on the adverse clinical outcomes in new statin users. The results suggested an association between statin adherence (measured by PDC) and decreased risk of MACE in patients who initiated statins for primary prevention of CVD, which are consistent with previous studies [11, 15–17, 23, 24]. But this trend was not observed among patients who initiated statins for secondary prevention of CVD, which may need further explorations. In the exploration of the relationship between statin adherence and risk of adverse clinical outcomes, dividing all new statin users into primary and secondary prevention subgroups separately is essential considering the heterogeneity of all statin users.

The results showed that statin adherence is very poor among new statin users both for primary and secondary prevention of CVD in China. The mean PDC of all statin users was 0.20, only 5.9% individuals with PDC ≥0.5, and less than 1% patients with PDC ≥0.8. A majority of statin users discontinued their statin treatment in the initial 3 months and didn’t restart anymore, which was the primary reason of the poor adherence. These estimates in China are much lower than the results found in other countries and regions such as in Canada, UK, Italy, Finland, Taiwan, et al., in which the proportion of statin users with PDC or MPR ≥0.8 ranged from 40.8 to 74.0% [9–12, 16, 17]. It was known that adherence to medications for the prevention of asymptomatic chronic diseases in real-world practice settings is suboptimal [25], such as the case for statins used for dyslipidemia [19]. However, it was unexpected that statin adherence was so poor in China. Research based on Chinese patients had found that ‘health literacy’ could be a factor that contribute to poor medication (including statins, aspirin, clopidogrel, b-blockers, etc.) adherence, which means that the patients felt they no longer need to take the medication if their conditions had improved [26]. This reflected Chinese patients’ irregular medication use behaviors and may explain the poor statin adherence in this study. Raising Chinese patients’ health literacy through social propaganda could be a way to improve statin adherence. Besides, previous research has shown that patients receiving polypill that combines multiple active pharmaceutical ingredients in one pill form, rather than single pills were more likely to be adherent [27]. Therefore, making patients and doctors transfer from single pill to polypill through appropriate reimbursement policies would be helpful to improve statin adherence too.

In this study, statin adherence (PDC ≥ 0.5) was associated with a 37% reduced risk of adverse clinical outcomes in patients who initiated statins for primary prevention of CVD in this study. In previous real-world studies, the benefits associated with adherence to statins (PDC ≥ 0.75 or 0.8 or 0.9) to reduce risk of adverse clinical outcomes (coronary artery disease, ischemic heart disease, cerebrovascular disease, death, etc.) in patients without CVD and using statins for primary prevention purpose have been observed and ranged from 18 to 42% [11, 15–17, 23, 24]. The relatively low adherence and finding between statin adherence and reduction in risk of MACE in this study highlighted the urgent need for effective strategies to increase Chinese patients’ statin adherence.

Previous studies also found statin benefit in reducing risk of adverse clinical outcomes such as MACE among patients for secondary prevention purpose, while this was not proved by the present study. The reduced risk of adverse clinical outcomes benefit from statin adherence (PDC ≥ 0.8) in previous studies ranged from 15% ~ 85%, depending on different samples included (such as stroke survivors, myocardial infarction survivors, acute coronary syndrome survivors, patients with coronary heart disease), and different adverse clinical outcomes measured (such as recurrence of ischemic stroke or myocardial infarction, acute coronary event, major adverse cardiovascular events, death) [8, 9, 20, 21, 28, 29]. Possible explanations for not detecting any relationship in secondary prevention subgroup in this study may include: (1) Patients in secondary prevention subgroup had different CVD and may also be heterogeneous with various disease severity within each disease; (2) Using 0.5 as a cutoff point of PDC may not be suitable enough to capture the benefit of statin adherence compared with 0.8. However, the sample size of patients with PDC ≥0.8 in this study was not enough for us to conduct the data analysis.

This study also had some limitations. Firstly, as mentioned above, PDC = 0.5 was used as the cutoff of adherence when exploring the association between adherence and risk of MACE, rather than PDC = 0.8, the commonly used adherence cutoff, which might lead to an underestimation of the benefits of statin adherence, and made the observed benefits not directly comparable to some other studies. However, a retrospective study using 0.2 as the interval of PDC to divide the subgroups found that patients with better statin adherence had lower risk of adverse clinical outcome, which suggested a dose response between statin adherence and risk of adverse clinical outcomes [10]. Therefore, considering the limited sample size of patients with PDC ≥ 0.8 in our study, choosing 0.5 as the cutoff of PDC might also have certain rationality. Secondly, excluding patients with MACE in the first 12-month follow-up period could lead to an immortal time period, which might cause an underestimation of risk of MACE. But no bias would be incurred for the comparison between patients with PDC ≥ 0.5 and patients with PDC < 0.5 as the same excluding criteria was used. Thirdly, adherence was estimated based on the prescriptions, but whether the patient actually took the medicine was uncertain. Fourthly, the overlaps of the prescriptions were dropped which may lead to an underestimate of PDC, but this impact could be negligible in our analyses since the mean PDC would only increase from 0.20 to 0.21 if they were not dropped. Finally, the analyses were based on UEBMI database that covered employees and retirees in Tianjin, and the rest of Tianjin residents including unemployed people, young children and students were covered by the other public basic medical insurance. Therefore, the results of this study might not be representative enough for all populations in China, and the relatively higher reimbursement ratio of UEBMI might lead to a higher statin adherence compared with other insurance programs.

## Conclusions

In conclusion, statin adherence was very poor both in primary and secondary prevention new statin users in China. The relationship between statin adherence and reduced risk of MACE was found in Chinese patients who initiated statins for primary prevention of CVD, but this relationship was not detected in patients who initiated statins for secondary prevention of CVD, which might need further explorations. Findings from this study highlighted the importance of improving statin adherence in Chinese patients.

## Data Availability

The data that support the findings of this study are available from Tianjin Municipal Human Resources and Social Security Bureau but restrictions apply to the availability of these data, which were used under license for the current study, and so are not publicly available. Data are however available from the authors upon reasonable request and with permission of Tianjin Municipal Human Resources and Social Security Bureau.

## Abbreviations

CVD: Cardiovascular diseases
PDC: Proportion of days covered
MPR: Medication possession ratio
MACE: Major adverse cardiovascular events
SD: Standard deviation
UEBMI: Urban employee basic medical insurance
CABG: Coronary artery bypass grafting
PCI: Percutaneous coronary intervention
CEA: Carotid Endarterectomy
CCI: Charlson comorbidity index
CKD: Chronic kidney disease
HR: Hazard ratios
PSM: Propensity score matching
